# Cluster of Donor-Derived Cryptococcosis after Liver and Kidney Transplantation

**DOI:** 10.3201/eid2810.220522

**Published:** 2022-10

**Authors:** Meng Sha, Chuan Shen, Ying Tong, Qiang Xia

**Affiliations:** Shanghai Jiao Tong University, Shanghai, China

**Keywords:** cryptococcosis, fungi, Cryptococcus neoformans, disease cluster, donor-derived infection, liver transplantation, kidney transplantation

## Abstract

Cryptococcosis infection after transplantation is easily overlooked or misdiagnosed. We report a cluster of donor-derived cryptococcosis infection in liver and kidney transplant recipients from the same donor in China. Infections occurred within 1 month after transplantation, and were confirmed by using biopsies and blood tests.

Cryptococcosis is the third most common invasive fungal infection in solid-organ transplants (*1**,**2*). The incidence of cryptococcosis in transplant recipients was estimated to be 0.76% in mainland China, and the *Cryptococcus neoformans* variant *grubii* genotype was the predominant species (*3**–**5*).

Cryptococcosis after transplantation is easily overlooked because of high diversity of clinical symptoms, which leads to mortality rates as high as 20% (*6*). Another feature of recipient-acquired cryptococcosis is the late onset of infection, which usually is 15‒21 months posttransplant (*7*). However, donor-derived transmission should be considered if disease is found within 1 month posttransplant or if multiple recipients from the same donor become ill (*8**,**9*). We report a cluster of donor-derived cryptococcosis after liver and kidney transplantation in China.

This study was approved by the Administration Committee of Shanghai Jiao Tong University, China. Written informed consent was obtained from the patient for the anonymized information to be published in this article.

The transplant donor was a 60-year-old man who had severe cerebral infarction, which progressed to brain death. Chest computed tomography (CT) scan showed clear lung fields and no infiltration. At organ procuring, the liver and kidney grafts looked grossly normal. Routine donor biopsy did not show any histopathologic abnormality. However, retrospective testing of donor serum for cryptococcal antigen (CrAg; Lateral Flow Assay; Immuno-Mycologics, Inc., https://www.immy.com) showed a titer of 1:8 seven days after grafts had been transplanted. One of 2 blood cultures at the time of organ procurement became positive after 8 days of incubation. *C. neoformans* was subsequently identified.

The first recipient was a 64-year-old man who had hepatocellular carcinoma and underwent liver transplantation. The transplant was successful, and there were no immediate complications. Postoperative aminotransferase levels decreased gradually. However, the recipient had progressive jaundice. The total bilirubin level increased from 103.6 μmol/L on postoperative day (POD) 1 to 704.3 μmol/L on POD 15. The patient had no fevers, cough, or dizziness.

Liver biopsy on POD 7 showed no evidence of rejection, biliary complications or drug-induced liver injury. The unexpected jaundice persisted and showed no major decrease. Liver biopsy was performed on POD 30. Large numbers of encapsulated yeasts were found inside the liver. Microscopically, the colonized organism had an oval shape and a loose surrounding histiocytic response (Figure). A subsequent recipient serum sample was positive for CrAg (titer >1:2,560) on POD 32.

**Figure:**
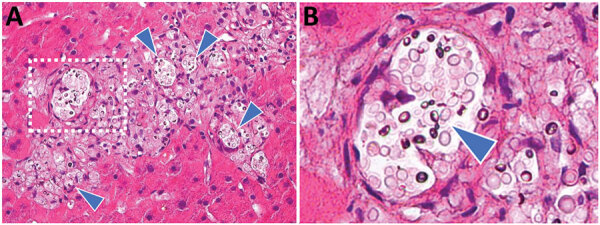
Transplanted liver tissue biopsy specimen on postoperative day 30 from donor in cluster of donor-derived cryptococcosis, China. A) Hematoxylin and eosin stain shows cryptococcal yeast liver (arrowheads). Original magnification ×200. B) Enlarged view of boxed area from panel A. Original magnification ×400.

The recipient received amphotericin B lipid complex plus 5‐flucytosine for 4 weeks. A gradual decrease in bilirubin was observed. The antifungal treatment was changed to oral fluconazole after he was discharged. Follow-up of CrAg showed a decrease from 1:2,560 to 1:32 at 1 year after transplant. Fluconazole was discontinued 15 months after transplant. The recipient showed good liver function for 30 months without active infection (Table). Hepatocellular carcinoma did not recur.

**Table:** Postoperative cryptococcal antigen titer change and antifungal treatment regimen for transplant donor in cluster of donor-derived cryptococcosis, China

Postoperative day	Cryptococcal antigen titer	Treatment
30	>1:2,560	Amphotericin B lipid complex and 5‐flucytosine
60	>1:1,280	Oral fluconazole, 400 mg/d
90	>1:640	Oral fluconazole, 400 mg/d
120	1:640	Oral fluconazole, 400 mg/d
180	1:128	Oral fluconazole, 400 mg/d
270	1:128	Oral fluconazole, 400 mg/d
360	1:32	Oral fluconazole, 400 mg/d
450	Negative result	Discontinued

The second recipient was a 65-year-old man who had end-stage renal disease and received a kidney transplant from the same donor. The graft function recovered uneventfully. The recipient was discharged on POD 6 and received an immunosuppression regimen of tacrolimus and mycophenolate. However, the recipient had a low fever and cough on POD 21. Chest CT showed pulmonary consolidations and infiltration. Bronchoalveolar lavage was not performed because intubation was not conducted; there were no signs of hypoxia. However, a CrAg titer of 1:1,280 and positive blood culture resulted in a diagnosis of cryptococcal pneumonia. Antifungal therapy was given for 4 weeks, and oral fluconazole maintenance therapy was given subsequently. The recipient recovered and showed standard graft function and no signs of infection.

The third recipient was a 50-year-old woman who received a kidney transplant from the same donor. She was discharged on POD 6 and had no specific complaints. On POD 26, she reported dizziness, diplopia, and severe headache and was readmitted to the hospital. Fluid from a lumbar puncture culture showed *C. neoformans*. The serum CrAg titer was >1:2,560. The recipient was given amphotericin B lipid complex and 5‐flucytosine. However, loss of consciousness and a convulsion occurred on POD 31. Further brain CT showed serious cerebral hemorrhage and compression of the brainstem. Her family withdrew care at that point, and the recipient died. Autopsy showed that the glomeruli of the transplanted kidney and spinal cord were infiltrated with oval-shaped yeast consistent with *C. neoformans*.

The recipients had negative clinical signs and no CrAg pretransplantation. However, the liver and kidney recipients who received organs from the same donor all showed development of cryptococcosis. *Cryptococcus* sp. in the blood culture and biopsies makes donor-derived transmission the most likely means of infection. Communication gaps between the microbiology laboratories and transplant team were associated with the donor-derived infection of our case. Positive blood culture results should be communicated immediately to initiate antifungal treatment promptly.

Although illnesses and deaths from donor-derived cryptococcosis remain high, results for these case-patients emphasize an increased pretransplant clinical awareness of donor-derived infection. Serum CrAg might identify infected donors and enable effective prophylaxis. In addition, timely communication of suspected results is critical to improve outcomes.
